# Plasmapheresis leading to remission of refractory nephrotic syndrome due to fibrillary glomerulonephritis: a case report

**DOI:** 10.1186/1752-1947-6-116

**Published:** 2012-04-24

**Authors:** Rainer U Pliquett, Peter Mohr, Badr El Din Mukhtar, Matthias Girndt, Silke Markau

**Affiliations:** 1 Department of Internal Medicine II, University Hospital Halle, Martin-Luther-University Halle-Wittenberg, Ernst-Grube-Str. 40, 06120 Halle (Saale), Germany; 2 Dialysis Center, Bahnhofstr. 12, 06217 Merseburg, Germany

## Abstract

**Introduction:**

Fibrillary glomerulonephritis (FibGN) is characterized by extracellular deposition of Congo red-negative microfibrils within the glomerular mesangium and leads to gross proteinuria or nephrotic syndrome. After diagnosis of FibGN, end-stage renal disease occurs within four years in 50% of patients.

**Case presentation:**

A 36-year-old Caucasian woman with proteinuria and intermittent nephrotic syndrome due to FibGN intermittently received immunosuppressive therapies, including glucocorticoids, mycophenolate mofetil, and rituximab, for 10 years. However, disease remission was not achieved and progressive kidney injury developed. Ultimately, in stage IV of chronic kidney disease (Kidney Disease: Improving Global Outcomes), three cycles of plasmapheresis of five to seven sessions each were performed every three to four months, reducing steady-state proteinuria from 7 to less than 1 g/day. Here, plasmapheresis led to a remission of nephrotic syndrome associated with FibGN.

**Conclusions:**

Plasmapheresis therapy is proposed as a further option for immunosuppressant-refractory FibGN.

## Introduction

Fibrillary glomerulonephritis (FibGN), a rare type of glomerulopathy, is characterized by extracellular deposition of Congo red-negative microfibrils (18 to 22 nm) within the glomerular mesangium and its capillary walls and leads to gross proteinuria or nephrotic syndrome. After diagnosis of FibGN, end-stage renal disease occurs within four years in 50% of patients [1]. Unlike amyloidosis, cryoglobulinemia, and light-chain glomerulopathy in patients with multiple myeloma, FibGN is a fibrillary glomerulopathy of unknown etiology. Viral infections such as hepatitis C [2] or human immunodeficiency virus (HIV) [3] have been suggested to cause FibGN.

There is no generally accepted therapeutic strategy for FibGN. Early on after diagnosis, FibGN is likely to be steroid-sensitive [4]. For steroid-refractory cases, rituximab [5] or cyclophosphamide [6] is a therapy alternative. Rituximab has yielded a partial reduction in nephrotic-range proteinuria due to FibGN [5], whereas cyclophosphamide has been reported to have an effect on nephrotic-range proteinuria in a case report only [7]. A therapeutic effect of cyclophosphamide in FibGN was lacking in a retrospective cohort study [8]. Concerning the use of mycophenolate as part of a combination or monotherapy in FibGN, limited experience exists. In four of 66 published FibGN cases, mycophenolate was used and a partial remission was achieved in two of them [9]. To the best of our knowledge, results of plasmapheresis therapy applied in cases of immunosuppressant-refractory FibGN have not been reported before. Here, we present a case of immunosuppressant-refractory nephrotic syndrome due to FibGN with remission after repeat plasmapheresis therapy.

## Case presentation

**Figure 1:**
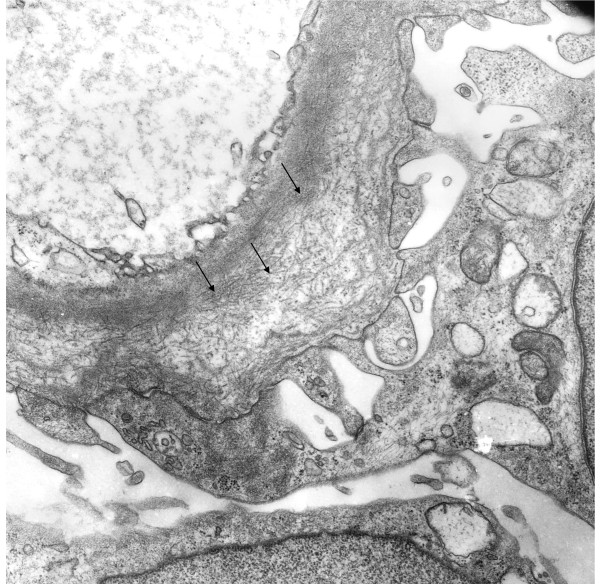
**Electron microscopy image of a capillary cross-section shows a portion of glomerular basement membrane with membranous deposits of 15- to 20-nm fibrils (arrows)**. Fibril density is highest in the vicinity of the capillary lumen and becomes lower toward the podocyte side of the glomerular basement membrane. Magnification: 12,000×.

A 36-year-old normotensive Caucasian woman was admitted to our nephrology department one year after a non-selective glomerular proteinuria of 2.1 g/day, leukocyturia (10/μL), and microhematuria were diagnosed. Comorbidity included allergic asthma, obesity with a body mass index of 46.9 kg/m^2^, and gastroesophageal reflux disease. Standard laboratory tests showed normoglycemia, glomerular hyperfiltration (creatinine clearance of 157 mL/minute), and an elevated leukocyte count (12,600 cells per microliter). Erythrocyte sedimentation rate was repeatedly found to be elevated (40/43 mm/hour). Except for a borderline anti-nuclear antibody titer (1:40) and smooth muscle antibody titer (1:80), the results of laboratory vasculitis screening were negative. On serological examination, active HIV or hepatitis (A, B, or C) infections were ruled out. Serum protein electrophoresis showed prominent alpha 1 (9.3%) and alpha 2 (13.2%) fractions and a double peak of the beta fraction. FibGN was established by typical electron microscopy findings in the renal biopsy (Figure 1). Upon diagnosis, an angiotensin-converting enzyme (ACE) inhibitor, diuretics, and a protein-restricted diet were initiated.

Six months after the diagnosis of FibGN, prednisolone (60 mg/day tapered off to 10 mg every other day) was added temporarily. However, proteinuria (2.5 g/day) did not improve.

Thirteen months later, nephrotic syndrome (proteinuria of 6.3 g/day) occurred after a respiratory infection. Estimated glomerular filtration rate (eGFR) [9] was 108.6 mL/minute per 1.73 m^2^. Both leukocyturia and microscopic hematuria deteriorated. Anti-nuclear antibody titer was 1:160, and leukocytosis (13,300 cells per microliter) persisted. Complement C3 and C4 were within normal range, and cryoglobulinemia was ruled out. Both plasma and urinary protein immunofixations were unremarkable. Abdominal ultrasound, gynecologic, pulmologic, and urologic exams yielded normal results. Cardiac workup, including echocardiography and electrocardiography (ECG), was unrevealing except for an incomplete right bundle branch block and intraventricular conductance abnormalities in ECG leads II and aVF. A second renal biopsy confirmed FibGN. The prednisolone dose was temporarily increased (60 mg/day for three months and tapered off to 5 mg every other day) and had to be discontinued after five more months because of side effects consistent with iatrogenic Cushing's syndrome (for example, weight gain, glucose intolerance, mood changes, and typical fat distribution). After discontinuation of steroids, proteinuria remained below 3 g/day for three years. However, over the course of a total of five years without immunosuppressive therapy, proteinuria worsened (4.8 g/day) and eGFR decreased to 64 mL/minute per 1.73 m^2^.

Six years after the diagnosis of FibGN, nephrotic syndrome recurred (proteinuria of 16.5 g/day) during a respiratory infection. Both steroid therapy (pulse and tapering off over the course of four weeks) and mycophenolate mofetil therapy (2 g/day for three months) were applied. During these therapies, proteinuria leveled off at 2 g/day whereas renal function remained impaired, indicative of chronic kidney disease: KDIGO (Kidney Disease: Improving Global Outcomes) stage III (eGFR of 50 mL/minute per 1.73 m^2^). Eight years after disease onset, our patient was readmitted for nephrotic syndrome (proteinuria of 20.8 g/day). Albumin/creatinine ratio (ACR) was 3.99 g/g. An aggravated arterial hypertension was addressed by switching the ACE inhibitor to a combination of the angiotensin receptor blocker valsartan and the direct renin antagonist aliskiren. In addition, our patient received rituximab (375 mg/m^2^) weekly for four weeks. In follow-up exams, proteinuria leveled off at 7 g/day (ACR of 2.98 g/g) whereas eGFR decreased to 32 mL/minute per 1.73 m^2^.

**Figure 2:**
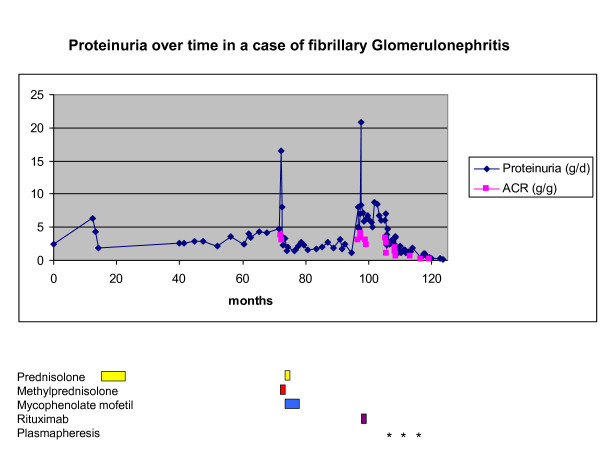
**Proteinuria over time in a case of fibrillary glomerulonephritis (FibGN)**. Proteinuria (in grams per day) and albumin/creatinine ratio (in grams of albumin in urine per grams of creatinine in urine) are displayed over time (in months) since the diagnosis of FibGN. Therapeutic interventions are specified below.

One year later (or nine years after disease onset), our patient complained of fatigue. The results of ECG and transthoracic echocardiography remained unchanged, thereby rendering a cardiac involvement [10] unlikely. Given the persisting gross proteinuria of 7 g/day, plasmapheresis was chosen as salvage therapy to decrease a putative plasma factor contributing to FibGN. Three cycles of five to seven plasmapheresis sessions each were performed during three hospital stays within nine months. During each session, 3 L of plasma or 71% of estimated plasma volume was replaced by a 5% human albumin solution. The treatment was well tolerated. Coagulation tests and immunoglobulin G checks were performed regularly. After the first cycle, proteinuria decreased from 7 to 2.2 g/day. After the second one, proteinuria decreased to 1.2 g/day. Finally, after the third cycle, proteinuria was below 1 g/day, indicating that nephrotic syndrome was in remission (Figure 2). Likewise, ACR decreased from 3.3 g/g before the onset of plasmapheresis therapy to less than 0.2 g/g after three cycles of plasmapheresis. Renal function remained impaired (eGFR of 23 mL/minute per 1.73 m^2^) once steady state was reached. Leukocyturia decreased from 140 to 7/μL and hematuria improved from 666 to 24 erythrocytes/μL after three cycles of plasmapheresis.

To maintain nephrotic syndrome remission, plasmapheresis sessions were scheduled to be performed every three months. In the maintenance phase, two intermittent plasmapheresis sessions were actually performed. However, the last one had to be interrupted after 45 minutes because of an allergic reaction to albumin in the exchange fluid. Thus, allergy prophylaxis will be considered in upcoming plasmapheresis sessions guided by proteinuria.

**Figure 3:**
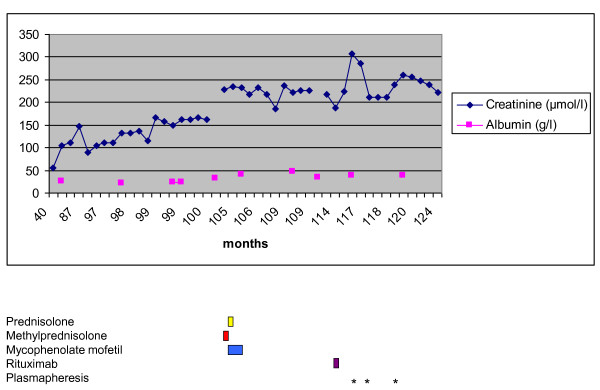
**Serum creatinine and albumin over time in a case of fibrillary glomerulonephritis (FibGN)**. Serum creatinine (in micromoles per liter) and serum albumin (in grams per liter) are displayed over time (in months) since the diagnosis of FibGN. Therapeutic interventions are specified below.

Ten months after induction plasmapheresis therapy (or 10 years after the onset of FibGN), remission of nephrotic syndrome prevailed. Residual proteinuria (0.2 g/day) and creatinine clearance (31.7 mL/minute, KDIGO stage III) remained stable. Figure 3 displays the course of serum creatinine and serum albumin. Following onset of plasmapheresis, our patient made a considerable recoveryin terms of quality of life, she resumed a part-time employment.

## Discussion

In this case of immunosuppressant-resistant nephrotic syndrome due to FibGN, plasmapheresis therapy was chosen as induction (three cycles or 18 sessions in total) and maintenance therapy, leading to a remission of nephrotic syndrome in terms of proteinuria reduction to less than 1 g/day. The already chronically impaired renal function remained stable. Hypothetically, respiratory infections triggered nephrotic syndrome episodes in this patient with FibGN. Before plasmapheresis therapy, FibGN-associated gross proteinuria or nephrotic syndrome was intermittently treated with glucocorticoids, mycophenolate mofetil, and rituximab in an escalating fashion over the course of 10 years. Glucocorticoids were not tolerated by our patient. Alternative immunosuppressive therapies only partially improved episodes of acutely worsened nephrotic syndrome. Long-term success has not been achieved. Finally, nephrotic-range proteinuria prevailed and renal function worsened.

Owing to the lack of proven benefit for nephrotic syndrome-complicated FibGN [9] and toxicity concerns in long-term use, the FibGN presented here was not treated with cyclophosphamide. Nonetheless, we consider this FibGN case to be immunosuppressant-refractory. Given the prospect of progressive kidney failure, plasmapheresis therapy was chosen as an individual experimental therapy.

Here, evidence arises in favor of plasmapheresis therapy in FibGN. Although a previous review [11] showed no therapeutic effect of plasmapheresis in three patients with nephrotic syndrome due to immunotactoid glomerulonephropathy, our case of FibGN showed that proteinuria decreased after the initial plasmapheresis cycles. Considering this observation, we propose plasmapheresis therapy as a possible rescue therapy for nephrotic syndrome in FibGN. Under plasmapheresis maintenance therapy (two sessions in 10 months), glomerular filtration rate did not deteriorate during follow-up. Most importantly, plasmapheresis-induced remission of nephrotic syndrome improved the quality of life in this patient with FibGN.

It is unlikely that short-term removal of soluble plasma constituents will sustainably alter fibril deposition. The removal of potentially aggravating factors may, however, preserve podocyte function and thereby lower the proteinuria. In addition, intermittent plasmapheresis sessions may be an effective maintenance therapy given the limitation of the short observation period of six months. Whether this finding extends to other patients with FibGN remains unclear. The good initial response to plasmapheresis seen in our patient stirs interest for further research on this topic. Larger register studies summarizing different treatment approaches in FibGN are desirable as a prerequisite for clinical randomized trials.

## Conclusion

Immunosuppressant-resistant nephrotic syndrome due to FibGN may respond favorably to plamapharesis induction and maintenance therapy. Further studies including clinical randomized trials are needed.

## Abbreviations

ACE: angiotensin-converting enzyme; ACR: albumin/creatinine ratio; ECG: electrocardiography; eGFR: estimated glomerular filtration rate; FibGN: fibrillary glomerulonephritis; HIV, human immunodeficiency virus; KDIGO: Kidney Disease: Improving Global Outcomes.

## Consent

Written informed consent was obtained from the patient for publication of this case report and any accompanying images. A copy of the written consent is available for review by the Editor-in-Chief of this journal.

## Competing interests

The authors declare that they have no competing interests.

## Authors' contributions

RUP conceived this case report. PM revised the manuscript. BEDM participated in data collection and added insight to data interpretation. MG and SM made substantial contributions to the interpretation of data and participated in revising the manuscript. All authors read and approved the final manuscript.
